# Clinical significance of plasma D-dimer in ovarian cancer: A meta-analysis

**DOI:** 10.1097/MD.0000000000007062

**Published:** 2017-06-23

**Authors:** Jiacong Wu, Ziyi Fu, Guangquan Liu, Pengfei Xu, Juan Xu, Xuemei Jia

**Affiliations:** a Nantong Maternity and Child Health Care Hospital, Nantong; b Nanjing Maternity and Child Health Medical Institute, Nanjing Maternity and Child Health Care Hospital, Obstetrics and Gynecology Hospital Affiliated to Nanjing Medical University, Nanjing; c Department of Obstetrics and Gynecology, Nanjing Maternity and Child Health Care Hospital, Obstetrics and Gynecology Hospital Affiliated to Nanjing Medical University, Nanjing, China.

**Keywords:** D-dimer, meta-analysis, ovarian cancer, overall survival, venous thromboembolism

## Abstract

**Background:**

D-dimer has been widely used for the diagnosis and prognosis of ovarian cancer, but there is still controversy on its prediction value of ovarian cancer.

**Objectives:**

To explore the clinical significance of plasma D-dimer level on ovarian cancer systematically.

**Methods:**

Using PubMed, Cochrane Library, and Web of Science libraries, all the relevant studies for the diagnostic and prognostic value of plasma D-dimer for ovarian cancer and the relationship between elevated D-dimer level and venous thromboembolism (VTE) risk of ovarian cancer were searched till May 30, 2016. Standardized mean difference (SMD), odds ratio (OR), hazard ratio (HR), and 95% confidence interval (CI) were appropriately pooled.

**Results:**

A total of 15 eligible studies involving a total of 1437 cancer patients were included. No significant association was found between high D-dimer level and overall survival of patients with ovarian cancer (HR 1.32, 95% CI: 0.90–1.95, *P*  =  .044). However, subgroup analysis indicated that the sample sizes could explain the heterogeneity between studies. And elevated D-dimer could predict increased risk of mortality when the sample sizes were >100 (HR 1.800, 95% CI: 1.283–2.523, *P*  =  .845). Besides, plasma D-dimer level was significantly higher in malignant ovarian cancer patients compared with benign controls (SMD 0.774, 95% CI: 0.597–0.951, *P*  =  .39), higher in advanced ovarian cancer patients (International Federation of Gynecology and Obstetrics [FIGO] classification III and IV) than in early stage ovarian cancer patients (FIGO classification I and II, SMD 0.611, 95% CI: 0.373–0.849, *P*  =  .442). And high D-dimer level indicated high VTE risk (OR 4.068, 95% CI: 2.423–6.829, *P*  =  .629) of ovarian cancer patients.

**Conclusion:**

The plasma D-dimer level in ovarian cancer patients can predict the changes that correlated with disease progression and the VTE risk. But its predictive value for the prognosis of ovarian cancer was significantly dependent on the sample sizes. More well-designed studies with large sample sizes are needed to validate and update the findings of present study.

## 1 Introduction

Ovarian cancer is the leading cause of deaths of female with gynecological malignancies worldwide.^[1]^ In 2012, an estimated 238,719 women were diagnosed with ovarian cancer and 151,900 of them died.^[2]^ In the United States, about 87% of the serous ovarian cancer patients were diagnosed at advanced stage.^[3]^ Lack of early predictive biomarkers is responsible for the high mortality rate. Until now, the most widely used biomarker for monitoring of ovarian cancer is carbohydrate antigen 125 (CA-125).^[4]^ Approximately 83% of the patients at advanced stage have CA-125 levels >35 U/mL.^[5]^ However, CA-125 is also elevated in a small proportion of people with endometriosis, pelvic inflammatory disease, pregnancy, hepatic cirrhosis, acute heart failure, tuberculosis, pancreatic cancer, lung cancer, liver cancer, and so on.^[6,7]^ Therefore, it has a relatively low positive predictive value and is usually not considered as an independent predictor.^[8]^ Besides, there is still controversy on its prognosis prediction value of ovarian cancer.^[9–11]^ Thus, to find other reliable biomarkers or the biomarkers which could be used in combination with CA-125 for general population screening, effective evaluation and prognosis prediction of ovarian cancer is long-expected.

The development of cancer is often accompanied with several complicated changes of homeostatic system.^[12]^ Patients with cancer especially in the advanced-stage often underlie a state of hypercoagulation and exaggerated fibrinolysis.^[13]^ Tumor cells can activate the clotting-fibrinolytic system and release various of procoagulant fibrinolytic markers and hemostatic factors,^[9]^ which in turn stimulate proliferation and differentiation of vascular endothelial cells to promote neoangiogenesis.^[14]^ This stimulation further contributes to tumor growth, invasion, metastasis, and recidivism.^[15–17]^ D-dimer, a signal of activated coagulation system and an end-product of fibrinogen,^[18]^ has been accepted as an useful diagnosis and prognosis parameter for several malignancies.^[19–21]^ Besides, D-dimer has been proved to be directly and positively correlated with CA-125,^[12,22]^ and the combination of CA-125 and D-dimer to differentiate benign from malignant ovarian tumors was better than single detection of either CA-125 or D-dimer.^[23]^ Studies have found that D-dimer level increased significantly in patients with ovarian cancer, but evidence was not sufficient as most studies included only small numbers of patients,^[24–26]^ and the diagnostic and prognostic significance of plasma D-dimer in ovarian cancer has not yet been systematically analyzed. So we conducted this meta-analysis to explain the role of plasma D-dimer for diagnosis and survival prediction of ovarian cancer. In addition, we also evaluated the relationship between elevated D-dimer level and venous thromboembolism (VTE) risk.

## 2 Materials and methods

### 2.1 Protocol

Meta analysis integrates patients’ information from the articles that had been approved by either ethics committee or institutional review board, so our meta-analysis did not include the patient’ consent and ethical approval. We conducted this meta-analysis in accordance with the Preferred Reporting Items for Meta-Analyses (PRISMA) statement.

### 2.2 Search strategy

An electronic search was carried out from inception up to May 30, 2016, for the association between plasma D-dimer levels and ovarian cancer, from the databases PubMed (http://www.ncbi.nlm.nih.gov/pubmed); Cochrane Library (http://www.cochranelibrary.com/); and Web of Science (http://apps.webofknowledge.com/UA_GeneralSearch_input.do?product=UA&search_mode=GeneralSearch&SID=W1xTxGB1nHdk2AAHedn&preferencesSaved=).

Searches were limited to human studies using the MeSH terms combined with free words of “ovarian cancer” and “D-dimer,” without language restriction. Furthermore, hand searches were performed for unindexed study selection.

### 2.3 Study selection

Studies were included in this meta-analysis if they covered one of the following elements: comparisons of plasma D-dimer levels between groups of early and advanced ovarian cancers or ovarian cancers and benign controls, relationship between plasma D-dimer levels and VTE risk of ovarian cancers, prognostic effect of plasma D-dimer on overall survival (OS) or progression-free survival.

Inclusion criteria: prospective or retrospective studies on patients with ovarian cancer by pathological diagnosis, plasma D-dimer level was detected before operation or chemotherapy, and data of non-English papers can be extracted from the English abstract. Exclusion criteria: review, meta-analysis, repeated studies, and severe outliers.

### 2.4 Data extraction and quality assessment

All data were extracted by 2 independent reviewers. Discrepancies were resolved by discussion or via the involvement of a third reviewer when necessary. The quality of each selected article in our analysis was assessed by Newcastle–Ottawa scale (NOS). The following data were extracted: the first author's name, publication year, study location, detection method of D-dimer, group, sample sizes, cutoff point of D-dimer, follow-up period, International Federation of Gynecology and Obstetrics (FIGO) stage, and the other clinical parameters.

The D-dimer value was presented as mean ± standard deviation (mean ± SD). The data using median and range or interquartile range were converted to mean ± SD by using the method reported before.^[27,28]^ Associations between D-dimer level and the outcome were presented as hazard ratios (HRs) and corresponding 95% confidence intervals (95% CIs). And the survival data presented as Kaplan–Meier curves or log-rank test were converted to HRs and 95% CIs using the method reported before.^[29–31]^

### 2.5 Statistical analysis

All analyses were completed with Stata V.12.0 (StataCorp, College Station, Texas). Heterogeneity between studies was calculated by *Q* and *I*^2^ test. If *I*^2^ ≤50%, then a fixed-effect model was used, otherwise, a random-effect model was adopted and a subgroup analysis was performed to find sources of the heterogeneity. Sensitivity analysis was carried out to exclude outliers. Publication bias was assessed by Begg funnel plots. *P* <.05 was considered statistically significant, except where otherwise specified. All statistical tests were 2-tailed.

## 3 Results

### 3.1 Literature search

**Figure 1:**
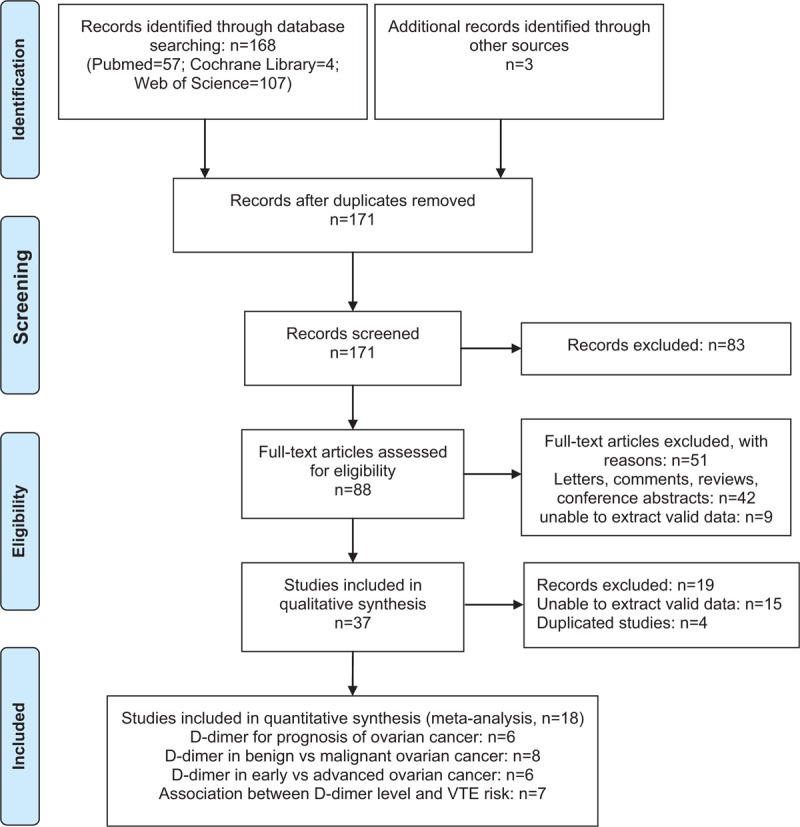
Flow chart of the study selection process.

The flowchart for the selection of literature is presented in Figure 1. In detail, a total of 171 records were returned (57 from PubMed, 4 from the Cochrane Library, 107 from Web of Science, and 3 from other sources). Eighty-three studies were excluded after the initial evaluation of titles and abstracts and 51 studies were excluded after the full-text reviewing. Besides, 15 studies were unable to extract the valid data and 4 duplicated studies were excluded. Finally, 18 studies with eligible data were included in this study. Six studies on the D-dimer level for the prognosis of ovarian cancer, 8 studies on the comparison of D-dimer levels between ovarian cancers (stages I–IV) and benign ovarian cysts, 6 studies on the comparison of D-dimer levels between early and advanced ovarian cancers, and 7 studies on the association between D-dimer level and VTE risk.

### 3.2 Study characteristics

The main characteristics of the studies included are listed in Table 1. All eligible articles were published from 1991 to 2016, and the sample sizes ranged from 30 to 241. In prognosis analyses and VTE risk analyses, each contained 1 study whose D-dimer level was detected after surgery. Quality assessment performed with NOS score is shown in Table 1 and the median score was 5 (range 4–8).

**Table 1:**
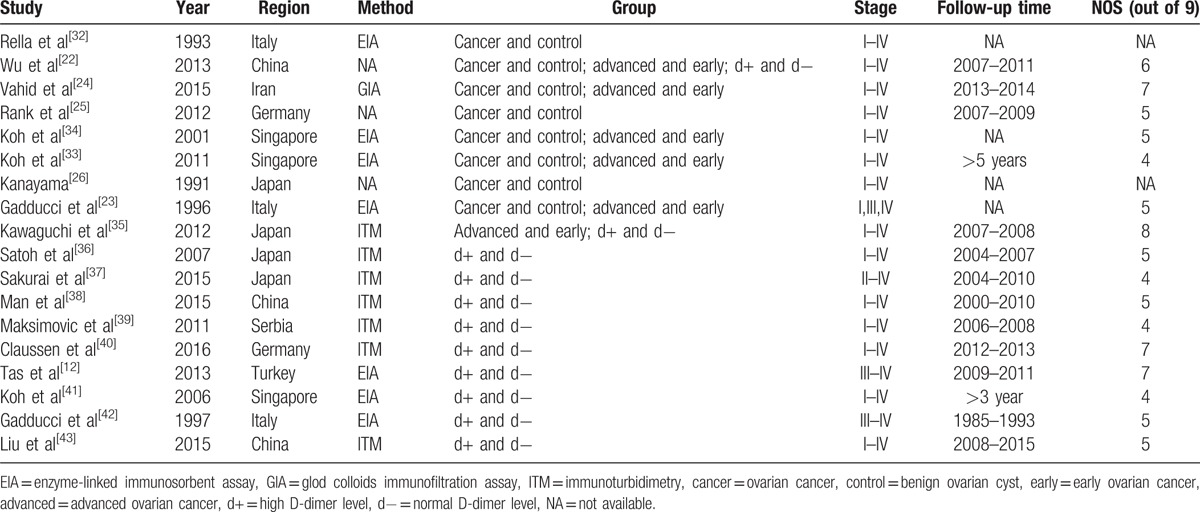
Study characteristics.

### 3.3 Main analysis

#### 3.3.1 D-dimer and prognosis

**Figure 2:**
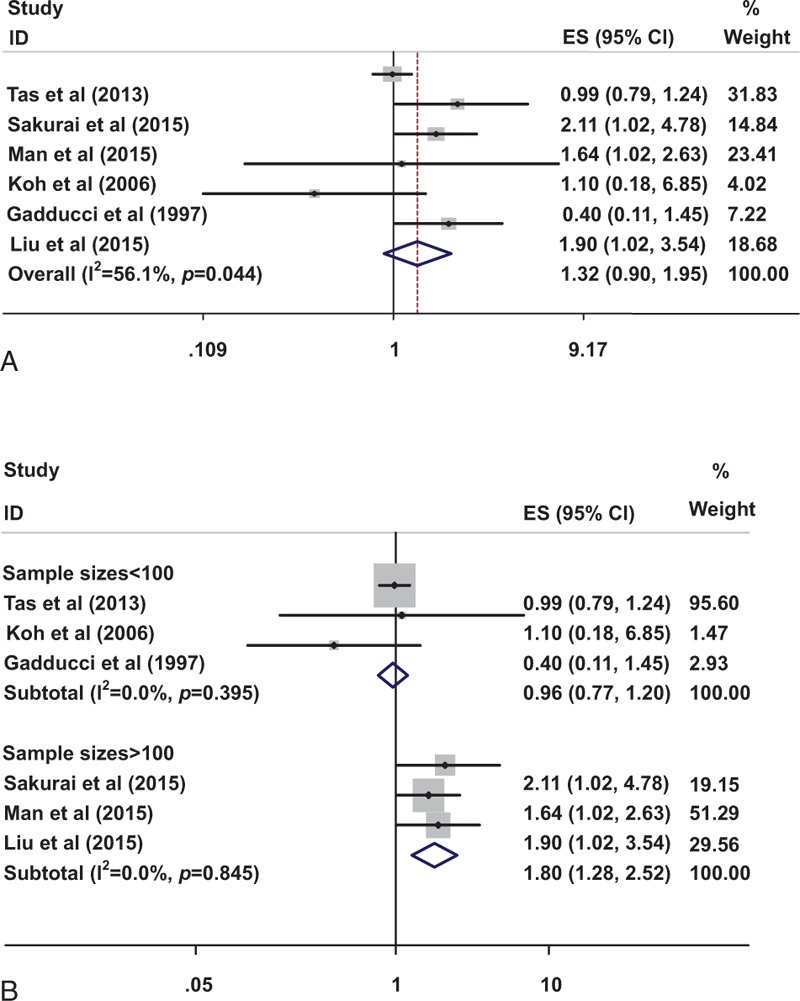
Impact of elevated D-dimer on OS (A) and subgroup analysis of different sample sizes on OS (B). OS  =  overall survival.

A total of 6 studies^[12,37,38,41–43]^ were focused on the D-dimer level on OS of ovarian cancer patients. The random-effect model with HR and 95% CI on OS indicated that there was no significant association between D-dimer and OS of ovarian cancers (HR 1.323, 95% CI: 0.897–1.953, Fig. 2A). However, the heterogeneity was high (*P*  =  .044, *I*^2^  =  56.1%).

A subgroup analysis stratified by the sample sizes showed that patients with elevated D-dimer had a relatively reduced risk of mortality compared with normal D-dimer group when the sample sizes were >100 (HR 1.800, 95% CI: 1.283–2.523), without evidence of heterogeneity (*P*  =  .845, *I*^2^  =  0.0%, Fig. 2B). However, no significant difference in OS was found in these two groups (HR 0.963, 95% CI: 0.771–1.201) when the sample sizes were <100 (*P*  =  .395, *I*^2^  =  0.0%, Fig. 2B). These results indicated that the small sample size may be the main source of heterogeneity.

#### 3.3.2 Comparison of the plasma D-dimer level in ovarian cancers (stages I–IV) and benign ovarian cysts

**Figure 3:**
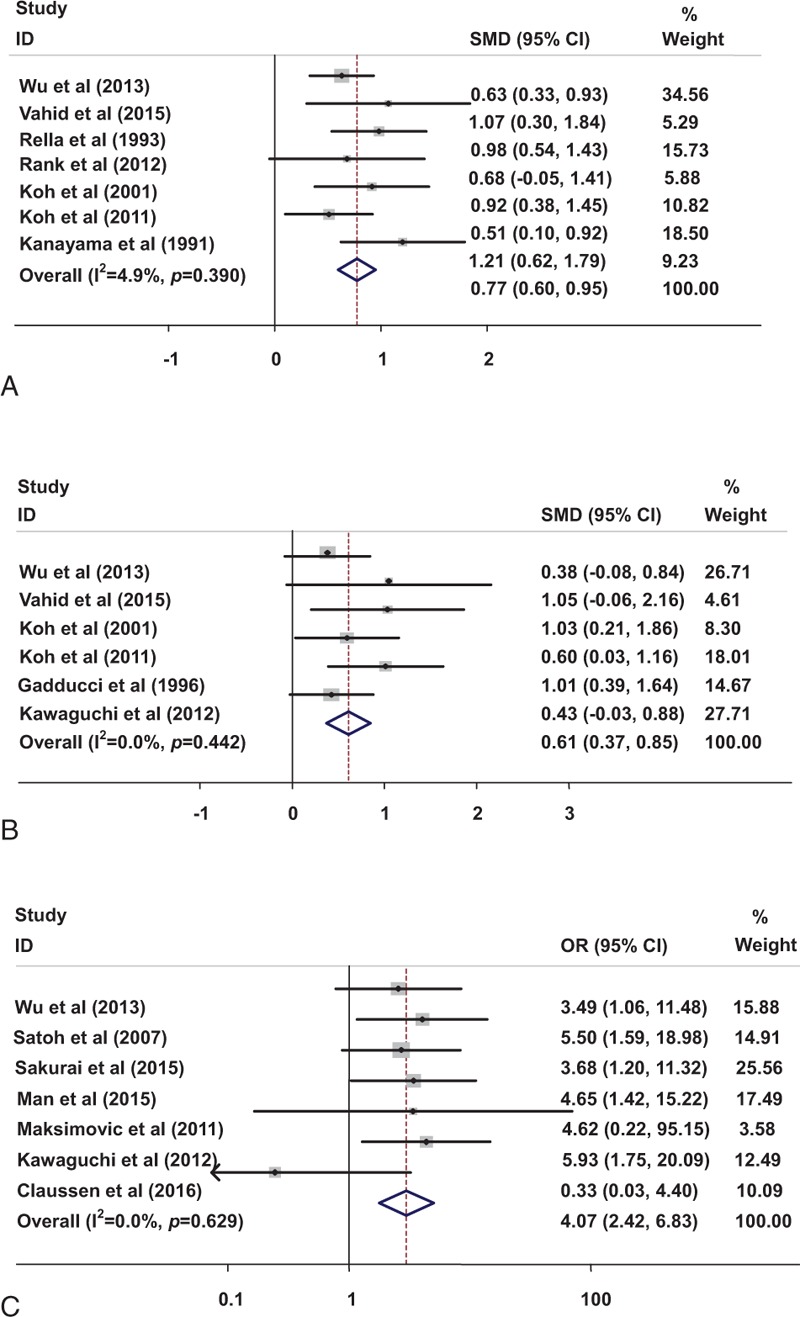
Forest plot for D-dimer in benign and malignant ovarian tumor (A), in early and advanced ovarian cancer (B). Risk of VTE associated with D-dimer level in ovarian cancer (C). VTE  =  venous thromboembolism.

A total of 7 studies^[22,24–26,32–34]^ involving 366 ovarian cancer patients and 233 benign controls investigated the association between D-dimer and ovarian cancer. Our results showed that patients with ovarian cancer (stages I–IV) showed a higher D-dimer level compared with benign controls (SMD  =  0.774, 95% CI: 0.597–0.951), with low degree of heterogeneity (*P*  =  .390, *I*^2^  =  4.9%, Fig. 3A) after the study by Gadducci et al^[23]^ was omitted (The sensitivity analysis showed that this paper lacked the data of FIGO stage II responsible for the heterogeneity (*P*  =  .000, *I*^2^  =  82.8%, data not shown).

#### 3.3.3 Comparison of the plasma D-dimer level in early ovarian cancers (stages I–II) and advanced ovarian cancers (stages III–IV)

With no evidence of heterogeneity (*P*  =  .442, *I*^2^  =  0.0%), a fixed-effect model was used in 6 studies^[22–24,33–35]^ to calculate the pooled SMD and 95% CI for the association of plasma D-dimer level with the FIGO stage of ovarian cancer. The results showed that the plasma D-dimer level was higher in advanced ovarian cancer patients (stages III–IV) than in the early stage ovarian cancer patients (stages I–II, SMD  =  0.611, 95% CI: 0.373–0.849; Fig. 3B).

#### 3.3.4 Risk of VTE in ovarian cancers with high D-dimer level and normal D-dimer level

The adjusted odds ratios (ORs) for 7 studies^[22,35–40]^ including the association between D-dimer level and VTE risk of ovarian cancer patients are shown in Fig. 3C. The results showed that the high D-dimer level was significantly associated with high VTE risk of ovarian cancer patients (OR: 4.068, 95% CI: 2.423–6.829), with no evidence of heterogeneity (*P*  =  .629, *I*^2^  =  0.0%).

### 3.4 Publication bias

**Figure 4:**
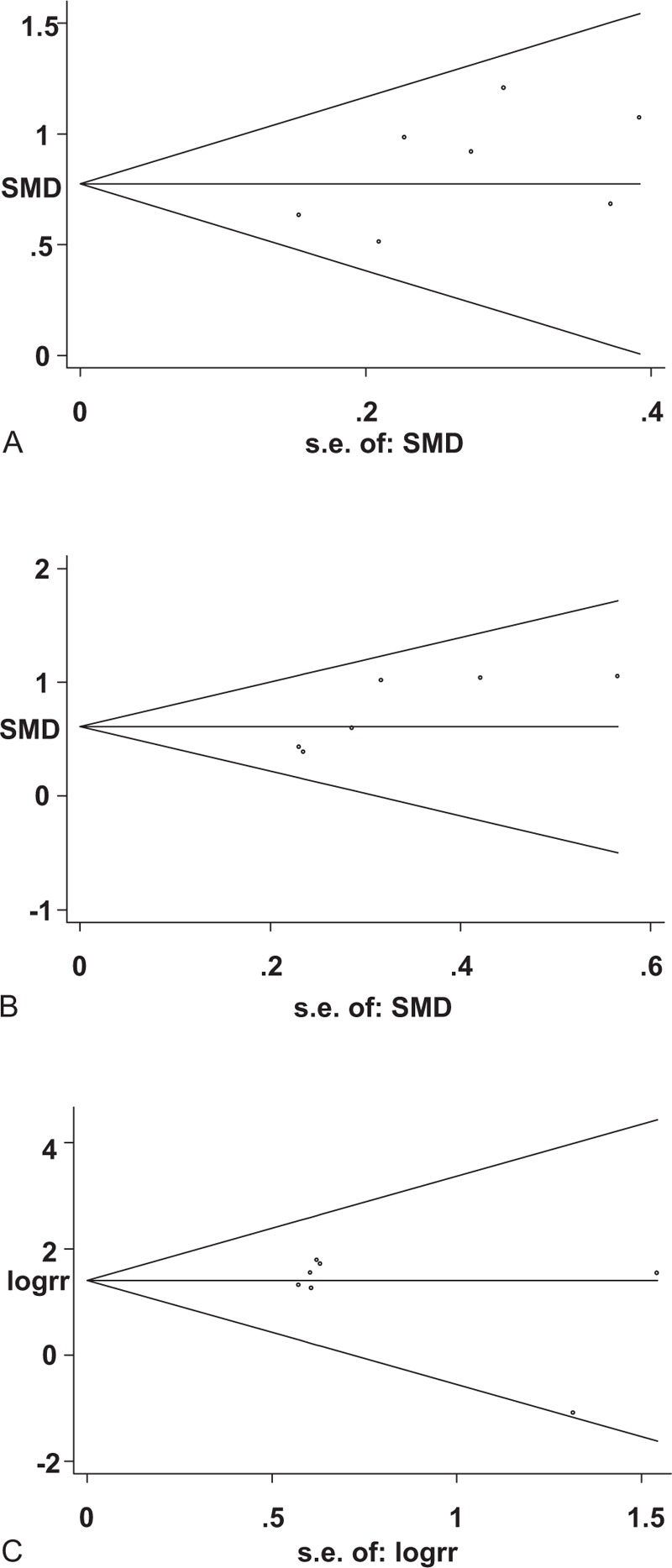
Begg's funnel plot for D-dimer in benign and malignant ovarian tumors (A), D-dimer in early and advanced ovarian cancers (B) and for VTE risk associated with D-dimer in ovarian cancer patients (C). VTE  =  venous thromboembolism.

The funnel plots showed no signs of asymmetry. And the *P*-value of the Begg rank correlation test (Fig. 4A–C, *P* >.05) showed that no publication bias existed among the included studies.

## 4 Discussion

D-dimer is a stable final product of fibrin degradation.^[44]^ Fibrinogen is converted into fibrin monomers by thrombin, and then the monomers polymerize to form fibrin clots during blood coagulation.^[17]^ Fibrin degradation products such as fragments E and D are released into the bloodstream after fibrin clots being digested by plasmin.^[14]^ Finally, D-dimers are formed steady with 2 covalently bound D-domains by factor XIII.^[18]^ D-dimer has been used as a specific marker which reflects the enhanced secondary fibrinolysis in fibrinolytic process.^[12]^ Its clinical value has been widely recognized, in particular for the exclusion of VTE.^[45]^ The adequate extra cellular matrix for cancer cell migration, invasion, and metastasis is provided from fibrin formation and remodeling process embodied as constantly elevated plasma D-dimer level.^[46]^ Studies^[17,21,22]^ indicate that the D-dimer level is positively correlated with the clinical stage of malignant tumor, which is consistent with the above-mentioned view.

A growing body of research focused on the relationship between D-dimer and tumors. A study of 32 women with breast cancer and 43 healthy controls reported that a high D-dimer level was correlated with poor outcomes of breast cancer.^[47]^ However, another study showed the opposite result.^[48]^ Studies on colorectal cancer^[49]^ and lung cancer^[21,50]^ patients indicated the association of elevated D-dimer with a high tumor stage. Now what about the ovarian cancer?

Our meta-analysis showed that the plasma D-dimer level in ovarian cancer patients was higher than in benign controls. And it was positively correlated with the FIGO classification. However, the sample sizes of the studies involving the comparison of D-dimer in early ovarian cancer and benign controls were too small and their results were not exactly the same^[22,23,33,34]^ or even opposite.^[24]^ Thus, we could not draw any conclusion to identify the diagnosis value of D-dimer in early stage ovarian cancer. Further studies that focus more on this topic are suggested. D-dimer level

The association between VTE and malignancy was first recognized by Trousseau in 1865.^[51]^ VTE includes deep vein thrombosis and pulmonary embolism and can significantly affect OS of patients.^[52]^ Up to now, quite a lot of studies demonstrated the high incidence of VTE in cancer patients,^[53–55]^ and they have confirmed that VTE was a common complication in malignant tumors.^[56]^ Interestingly, the tumors with high risk of VTE are mostly abdominal tumors which suggest that the mechanical stress in the complex abdominal venous system is easy to cause venous embolism.^[13,57]^ Ovarian cancer is one of the malignancies with the highest risk of VTE.^[57]^

Based on the formation mechanism, D-dimer has been used as a noninvasive screening index. The predictive value of D-dimer for VTE following ovarian cancer was still controversial.^[38–40]^ Our results showed that ovarian cancer patients with elevated D-dimer had a significant higher risk of VTE compared with normal D-dimer group. In other words, plasma D-dimer test may help to select occult VTE at the high-risk group with ovarian cancer. However, previous studies indicated that D-dimer test was proposed for exclusion rather than for diagnosis.^[35,36]^ The sensitivity of D-dimer for diagnosis and prognosis of ovarian is affected mainly by the sample sizes, the cutoff value, and the testing method.

This meta-analysis on prognostic value of D-dimer showed that OS was significantly reduced in patients with elevated D-dimer when the sample sizes were >100. However, no statistical difference were found between high and normal D-dimer level in terms of OS when sample sizes were <100. Besides, we also noticed that the cutoff point selection and the D-dimer detection method were also different between the large sample size group and small sample size group. Both cutoff point selection and D-dimer detection method are very essential for the evaluation of D-dimer level on OS. So we cannot conclude which part is the main source of heterogeneity. However, we consider that the cutoff point should be chosen according to the area under an ROC curve, but not just the median value. And the standard detection method should be recommended in the future.

As stated above, a lot of research has been done on this approach to detect tumor by using D-dimer as a marker, but its clinical use is limited possibly because of the following reasons. There are no unified testing methods of D-dimer and all standardization and harmonization attempts have not achieved satisfying results in daily practice.^[58]^ Differences in antibody specificities of different assays result in inconsistent quantitative determination in plasma samples.^[18]^ In conclusion, this meta-analysis indicates that plasma D-dimer can be used as a predictive marker in ovarian cancer and high D-dimer is related to subsequent VTE risk. Yet, there is insufficient evidence to support a significant association between elevated D-dimer and worse prognosis of ovarian cancer patients. Further researches are also required to confirm the value of D-dimer in the early diagnosis of ovarian cancer. Furthermore, multivariate models are needed to explore the independent effect of D-dimer on prognostic analysis.
